# The epidemiologic characteristics and associated risk factors of preterm birth from 2004 to 2013 in Taiwan

**DOI:** 10.1186/s12884-020-02903-1

**Published:** 2020-04-06

**Authors:** Yu-Kang Chang, Yuan-Tsung Tseng, Kow-Tong Chen

**Affiliations:** 1 Department of Radiology, Chi Mei Medical Center, Liouying, Tainan, Taiwan; 2 Department of Medical Research, Tainan Municipal Hospital (managed by Show Chwan Medical Care Corporation), Tainan, Taiwan; 3 Department of Occupational Medicine, Tainan Municipal Hospital (managed by Show Chwan Medical Care Corporation), No. 670, Chongde Road, East District, Tainan, Taiwan; 4 Department of Public Health, College of Medicine, National Cheng Kung University, Tainan, Taiwan

**Keywords:** Preterm birth, Spontaneous, Indicated preterm birth, Epidemiology

## Abstract

**Background:**

The rate of preterm birth has been increasing worldwide. Most preterm babies are at an increased risk of central nervous system impairments as well as respiratory and gastrointestinal complications. The aim of this study was to investigate the epidemiologic characteristics of and associated factors contributing to preterm birth in Taiwan.

**Methods:**

Information on obstetric antecedents and risk factors for preterm birth in pregnant women was obtained from the National Health Insurance Research (NHIR) database provided by the Taiwan National Health Research Institute. All live births from 2004 to 2013 in Taiwan were included in this study.

**Results:**

A total of 130,362 live births from 2004 to 2013 were included in this study. Overall, the average annual rate of preterm births increased by 5.3% (from 3.33% in 2004 to 5.11% in 2013). Multiple logistic regression analyses showed that nulliparous women, multifetal pregnancies, advanced mother age, history of preterm birth, history of maternal drug abuse/dependence, and maternal medical complications were positively associated with an increased risk of preterm birth (all *p-*values< 0.05).

**Conclusion:**

The overall proportion of preterm births increased from 2004 to 2013 in Taiwan. Babies born preterm had a higher risk of developing morbidities and mortalities. The development of a comprehensive program to identify the high-risk group is needed for effective interventions to prevent premature birth.

## Background

The trend of preterm birth rate has increased globally, despite introduction of the significant public health and medical prevention strategies for reducing the burden of preterm birth [1–3]. The rates of preterm birth are 12–15%, 5–9%, and 4.7–18.9% in USA, European countries, and China, respectively [4–7]. Preterm birth occurs for a variety of causes [8–10]. Several maternal demographic characteristics (e.g. advanced maternal age) and obstetric complications (e.G. *placenta* previa) are identified as a risk factor related to the occurrence of preterm birth [11–15].

Preterm birth is an important cause of long-term morbidity and death in children under 5 years of age globally [16–18]. Compared to infants born full term, preterm infants who do survive have a higher risk of neurodevelopmental impairments as well as respiratory and gastrointestinal complications [19]. Therefore, the development of effective preventive measures to decrease the morbidity of preterm birth is needed. An understanding of the epidemiology of preterm birth as well as the risk factors related to preterm births is required for the prevention of preterm labor. In this study, we planned to investigate the epidemiologic features of preterm births and determine the associated risk factors of preterm birth in Taiwan.

## Methods

### Data source

The study methods have been published previously [20, 21]. Briefly, this study is based on data from the National Health Insurance Research (NHIR) database provided by the Taiwan National Health Research Institute. The National Health Insurance (NHI) program in Taiwan was implemented in March 1995. As of 2007, as many as 96% of the citizens in Taiwan had joined the NHI program [20]. We collected a subset of data from the NHIR database with one million random subjects, accounting for ~ 5% of all subjects enrolled in the National Health Insurance program. The NHIR database contains every medical claim record (inpatient care and outpatient care), including age of mother, gravity, methods of delivery, medical disorders of mother, previous pregnancy complications, neonatal status, and the International Classification of Disease, Ninth Revision, Clinical Modification (ICD-9-CM) diagnosis [22].

### Study subjects

All babies alive starting at 20 weeks of gestation in Taiwan from 2004 to 2013 were included in the study. We excluded stillbirths (0.5%) and subjects with missing obstetric information (2.7%).

This work was approved by the Ethics Board, National Cheng Kung University Hospital, Tainan, Taiwan.

### Definition

Preterm birth was defined as a baby born alive before 37 weeks of pregnancy are completed [23, 24].

Spontaneous preterm birth was defined as babies born with spontaneous initiation of labor and prelabor rupture of membranes (PROM). Provider-initiated preterm birth was defined as babies born with provider-initiated mode of onset, either medical labor induction or cesarean delivery before the initiation of labor.

Gestational age was calculated on the basis of the last menstrual period (LMP) confirmed by sonographic examination prior to 20-week gestation [25, 26]. If the LMP and date by sonographic examination differed by more than 14 days, date by sonographic examination was used as the gestational age.

A first pregnancy woman who had never given birth was classified as nulliparity, and a woman who had borne 2 or more children was classified as multiparity [27]. Maternal age was calculated on the basis of mother age at the time of delivery [28].

Maternal medical disorders were defined as mother with existing medical diseases (e.g. hypertension), as well as obstetric complications (e.G. *placenta* abruptio) during the pregnancy period [21, 24].

### Ethical statement

We used the NHIR database for this study. According to the regulations of the Ministry of Health and Welfare, Taiwan, all of the NHIR datasets are only available from the Information Center, Ministry of Health and Welfare, Taiwan. This study was conducted according to the Declaration of Helsinki and was approved by the Institutional Review Board of National Cheng Kung University Hospital, Tainan, Taiwan. Informed consent was waived because all data in this study were unidentifiable and encrypted. The rights and welfare of the study subjects were not affected.

### Statistical analysis

We verified data, examined for their consistency, and removed missing values of data after data were collected. Univariate and multivariate analyses were performed in this study. The trend of preterm births was determined by calculating yearly proportions and displayed using a bar chart. Chi-squared test or Fisher exact test was used to determine the relationship between categorical variables and preterm birth and t-test was used for continuous variables. We used multivariable logistic regression to estimate preterm birth adjusted odds ratios (AOR) and their 95% confidence intervals (CIs) by maternal obstetric characteristics. An alpha value of 0.05 or less was considered as statistical significance. All statistical analyses were performed using SAS software version 9.4 (SAS Institute Inc., Cary, NC, USA).

## Results

Between 2004 and 2013, a total of 2,279,720 lives birth at greater than 20 weeks gestation were registered in Taiwan (www1.stat.gov.tw/ct.asp?xItem=15409&ctNode=4693&mp=3). In this study, 130,362 (5.7%) live births at greater than 20-week gestation being collected from NHIR database in Taiwan were enrolled in the study. Compared to the mean age of total population of live births in Taiwan, the mean age of our study subjects had not significant difference (31.2 years vs. 31.4 years; *p* > 0.05). Table 1 provides the descriptive statistics on our study subjects from 2004 to 2013. Among them, 124,393 (95.4%) were full-term births, 5969 (4.6%) were preterm births (5010 were spontaneous preterm births, and 959 were provider-initiated preterm births). According to parity, 66,224 (50.8%) were nulliparous, and 64,138 (49.2%) were multiparous. According to the gestational age, 130 (0.1%) babies were born at 20–27 weeks, 262 (0.2%) babies were born at 28–31 weeks, 5577 (4.3%) babies were born at 32–36 weeks, and 124,393 (95.4%) babies were born at > 37 weeks. According to the maternal age, 427 (0.3%) mothers were less than 20 years old at the time of delivery, 111,401 (85.5%) mothers were 20–39 years old, and 18,534 (14.2%) mothers were ≥ 40 years old. According to the multiplicity, 129,392 (99.3%) were singleton births, and 970 (0.7%) were multiple births. Among the study women, 221 (0.2%) were reported to have history of preterm birth, 230 (0.2%) were reported to have drug abuse/dependence, and 7143 (0.5%) were reported to have maternal medical complications.

**Table 1:** Demographic characteristics of live births enrolled in the study according to maternal obstetric characteristics in Taiwan, 2004–2013

Variables	Number *N* = 130,362	%
**Status of delivery**		
Full term	124,393	95.4
Preterm	5969	4.6
*Spontaneous preterm birth*	*5010*	
*Provider-initiated preterm birth*	*959*	
**Parity**		
Nulliparous	66,224	50.8
Multiparous	64,138	49.2
**Gestational age (weeks)**		
20–27	130	0.1
28–31	262	0.2
32–36	5577	4.3
≥ 37	124,393	95.4
**Maternal age (years)**		
< 20	427	0.3
20–39	111,401	85.5
≥ 40	18,534	14.2
**Multiplicity**		
Singleton birth	129,392	99.3
*Full term*	*123,797*	
*Preterm*	*5595*	
Multifetal births	970	0.7
*Full term*	*596*	
*Preterm*	*374*	
**History of preterm birth**	221	0.2
**History of maternal drug abuse/dependence**	230	0.2
**Maternal medical disorders**	7143	0.5

**Fig. 1:**
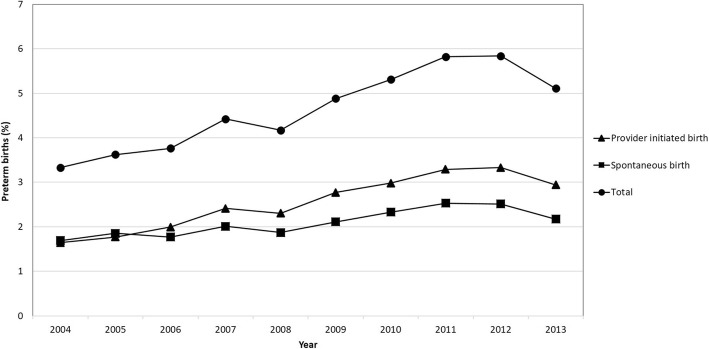
The trend of preterm birth among live births in Taiwan. 2004–2013

Figure 1 displays the yearly proportion of the preterm birth in Taiwan from 2004 to 2013. The proportion of preterm births increased over time (chi-square for linear trend = 199.4; *p* <  0.0001). The overall average preterm proportion was 4.7%. The average annual preterm birth rate steadily increased by 5.3% during the study period (from 3.33% in 2004 to 5.11% in 2013). The lowest rate was in 2004 (3.33%) and the highest rate was in 2012 (5.84%). When we stratified preterm births in population by spontaneous preterm and provider-initiated preterm birth, the change in the preterm birth rate after the spontaneous onset of labor for the total population was 0.5% (1.69% in 2004 to 2.17% in 2013), and the change in the preterm birth rate after provider initiated preterm for the total population was 1.3% (1.64% in 2004 to 2.94 in 2013).

Table 2 shows the potential risk factors of preterm births according to the univariate analysis. Compared to full-term births, the preterm-birth group had a higher rate of nulliparous women, multifetal births, advanced mother age (≥40 years), history of preterm birth, history of maternal drug abuse/dependence, and maternal medical complications.

**Table 2:** Univariate analysis of potential risk factors for preterm births in Taiwan, 2004–2013

Variables	Preterm (%) *N* = 5969	Full-term (%) *N* = 124,393	*P*-value
**Parity**			< 0.0001
Multiparous	2767 (46.4)	61,371 (49.3)	
Nulliparous	3202 (53.6)	63,022 (50.7)	
**Multiplicity**			< 0.0001
Singleton	5595 (93.7)	123,797 (99.5)	
Multifetal births	374 (6.3)	596 (0.5)	
**Maternal age (years)**			< 0.0001
< 20	19 (0.3)	408 (0.3)	
20–39	4655 (78.0)	106,746 (85.8)	
≥ 40	1295 (21.7)	17,239 (13.9)	
**History of preterm birth**			< 0.0001
No	5897 (98.8)	124,244 (99.9)	
Yes	72 (1.2)	149 (0.1)	
**History of maternal drug abuse/dependence**			0.0002
No	206	124,187	
Yes	24	5945	
**Maternal medical disorders**			< 0.0001
No	5050 (84.6)	118,169 (95.0)	
Yes	919 (15.4)	6224 (5.0)	

Table 3 we display the association between maternal demographic characteristics, obstetric complications, and the occurrence of preterm birth by the multivariate logistic regression analysis in Taiwan, 2004–2013. Significant contributors to preterm birth included nulliparous women (AOR 1.13; 95% CI 1.07–1.19; *p* <  0.0001), multi-fetal births (AOR 12.42; 95% CI 10.79–14.31; *p* < 0.0001), advanced maternal age (≥40 years) (AOR 1.61; 95% CI 1.01–2.56; *p* = 0.043), history of preterm births (AOR 10.18; 95% CI 7.68–13.50; *p <* 0.0001), history of maternal drug abuse/dependence (AOR 2.43; 95% CI 1.59–3.72; *p* = 0.0003), and history of maternal medical disorders (AOR 2.27; 95% CI 1.91–2.63; *p* < 0.0001).

**Table 3:** Multiple logistic regression analysis of risk factors for preterm birth in Taiwan, 2004–2013

Variables	AOR	95% CI	*P*-value
**Parity**			< 0.0001
Multiparous	1.00		
Nulliparous	1.13	1.07–1.19	
**Multiplicity**			< 0.0001
Singleton	1.00		
Multifetal births	12.42	10.79–14.31	
**Maternal age (years)**			0.043
< 40	1.00		
≥ 40	1.61	1.01–2.56	
**History of preterm birth**			< 0.0001
No	1.00		
Yes	10.18	7.68–13.50	
**History of maternal drug abuse/dependence**			0.0003
No	1.00		
Yes	2.43	1.59–3.72	
**Maternal medical disorders**			< 0.0001
No	1.00		
Yes	2.27	1.91–2.63	

*CI* confidence interval, *AOR* adjusted odds ratio; All odds ratios were mutually adjusted for other variables in the table

## Discussion

Our study, using the NHIR database, provides epidemiologic characteristics and associated risk factors for preterm births in Taiwan. This study highlighted a rising trend for preterm birth rate in Taiwan from 2004 to 2013. This finding was consistent with those of previous studies [29, 30].

We found that provider-initiated preterm delivery was positively associated with the occurrence of preterm birth in this study. This finding is similar to the findings in previous studies in the USA and Latin America [31–33]. This observation may be due to the higher accessibility to hospitals in Taiwan [34]. Taiwan is a small island and most of Taiwan residents (> 96%) are covered by the NHI program. Another possibility is medical doctors are increasingly more aggressive to manage the medical complications [35].

After adjusting for potential confounders, nulliparous women had a positive association with a higher risk of preterm delivery, which is compatible with previous studies [28, 36]. It has been reported that both advanced maternal age and multiparity are associated with higher risk of preterm birth [26, 37]. However, some studies comparing nulliparity and multiparity [38, 39] have suggested that advanced maternal age influences the risk of preterm birth, regardless of parity.

Previous studies have described that multifetal pregnancies are an important risk factor for preterm delivery [24, 40]. Multifetal pregnancies will have higher rate of spontaneous delivery or preterm birth due to PROM before 37 weeks of gestation [41]. Compared with a single fetus, some studies have indicated that the preterm birth rate was higher in the condition with multiple fetuses [42, 43]. Uterine overdistension may be the reason to explain why the risk of spontaneous preterm birth increased in women with multifetal pregnancies [40]. Similar to the previous study [40], our study found that multifetal pregnancies had a higher preterm birth rate.

Previous studies have also reported that advanced maternal age is positively associated with the risk of early preterm birth [44, 45]. Claramonte Nieto et al. [46] showed that maternal age was an independent risk factor for adverse obstetric outcomes and that maternal age ≥ 40 years was associated with relevant increased risks of adverse obstetric outcomes. In our study, we found that the risk of preterm birth was positively associated with advanced maternal ages (> 40 years). Another study found that maternal obesity was significantly associated with the risk of preterm birth according to maternal age and race or ethnicity [47]. These findings indicated that the higher risk in the preterm birth may be due to age-related factors (e.g., provider-initiated preterm births, chronic diseases) during the study period.

It was reported that more than 70% of all preterm births occurred at 32–36 weeks of gestation in previous study [48]. We found that the preterm birth rate was highest at 32–36 weeks gestation in Taiwan population (Table 1), which is compatible with previous studies [10, 48]. The mechanisms for the high prevalence of preterm birth at 32–36 weeks of gestation are unclear [49]. However, more people accessed the medical help by the reproductive technologies and more aggressive in perinatal management and neonatal intensive care could be the potential risk factors [50]. Therefore, fetuses at risk for death or genetic aberration could be screened out earlier, causing more preterm births at 32–36 weeks of gestation. In this study, we did not collect the information of using reproductive technology in the study population.

Consistent with prior studies [25, 28], our study also found that maternal medical complications and a history of preterm birth were associated with an increased risk of preterm birth. It has been estimated that the probability of preterm birth is increased as women have more history of preterm birth [51]. This finding may indicate that there is a certain genetic basis for preterm birth [12]. However, until now, no definite genotyping or markers were identified. Advanced biotechnology and analytical strategies are needed to explore the relationship between complex environmental and genetic factors. Understanding these factors and their interactions could lead to major improvements in the diagnosis, prevention, and treatment of preterm birth.

Previous studies showed that pregnant drug users had a significantly higher risk of preterm birth [52–54]. Our study also found that women with reported drug abuse/dependence during pregnancy had a higher risk of having a preterm birth compared with women without reported drug abuse/dependence.

### Strengths and limitations

The strength of this study was its large sample size with medical records obtained from the NHIR database. Despite this strength, several limitations were noted in this study. First, because this dataset was collected through a passive reporting system, underreporting or misclassification of obstetric information of study subjects would have occurred. Second, the estimates of risk for preterm deliveries generated by logistic regression analysis of data in this retrospective study should be interpreted cautiously, especially when the effect sizes of some risk factors were small and the confidence interval was wide. Third, information on certain risk factors, for example, maternal smoking history, BMI, or education level were insufficient in this database.

## Conclusions

In conclusion, this study highlighted that the preterm birth rate was rising in Taiwan between 2004 and 2013. The main predictive factors for the risk of preterm birth are nulliparous women, multifetal pregnancies, advanced maternal age (≥40 years), history of preterm birth, and maternal medical disorders. Babies born prematurely have a higher risk for the development of morbidities and mortalities. Therefore, developing a comprehensive program for identifying pregnancies at high-risk for preterm birth is needed for effective interventions to prevent premature births.

## Data Availability

The data that support the findings of this study are available from the National Health Institute, Ministry of Health and Welfare, Taiwan, but restrictions apply to the availability of these data, which were licensed for use in the current study; therefore, they are not publicly available. Data are, however, available from the authors upon reasonable request and with permission of National Health Institute, Ministry of Health and Welfare, Taiwan.

## Abbreviations

AOR: Adjusted odds ratio
BMI: Body mass index
CI: Confidence interval
ICD-9-CM: International Classification of Disease, Ninth Revision, Clinical Modification
LMP: Last menstrual period
NHI: National Health Insurance
NHIR: National Health Insurance Research
PROM: Prelabor rupture of membranes

## References

[1] Kenny LC, Lavender T, McNamee R, O'Neill SM, Mills T, Khashan AS (2013). Advanced maternal age and adverse pregnancy outcome: evidence from a large contemporary cohort. PLoS One.

[2] Goldenberg RL, Rouse DJ (1998). The prevention of premature birth. N Engl J Med.

[3] Tracy SK, Tracy MB, Dean J, Laws P, Sullivan E (2007). Spontaneous preterm birth of live born infants in women at low risk in Australia over 10 years: a population-based study. BJOG..

[4] Slattery MM, Morrison JJ (2002). Preterm delivery. Lancet..

[5] Shennan AH, Bewley S (2006). Why should preterm births be rising?. BMJ..

[6] Blondel B, Supernant K, Du Mazaubrun C, Breart G (2006). Trends in perinatal health in metropolitan France between 1995 and 2003: results from the National Perinatal Surveys. J Gynecol Obstet Biol Reprod (Paris).

[7] Han W, Song J, Liu A, Huo K, Xu F, Cui S, Wang X, Zhu C (2011). Trends in live births in the past 20 years in Zhengzhou, China. Acta Obstet Gynecol Scand.

[8] Savitz DA, Blackmore CA, Thorp JM (1991). Epidemiologic characteristics of preterm delivery: etiologic heterogeneity. Am J Obstet Gynecol.

[9] Goldenberg RL, Culhane JF, Iams JD, Romero R (2008). Epidemiology and causes of preterm birth. Lancet..

[10] Frey HA, Klebanoff MA (2016). The epidemiology, etiology, and costs of preterm birth. Semin Fetal Neonatal Med.

[11] Schemf AH, Branum AM, Luskacs SL, Schoendorf KC (2007). Maternal age and parity-associated risks of preterm birth: differences by race/ethnicity. Paediatr Perinat Epidemiol.

[12] Blencowe H, Cousens S, Oestergaard MZ, Chou D, Moller AB, Narwal R, Adler A, Vera Garcia C, Rohde S, Say L, Lawn JE (2012). National, regional, and worldwide estimates of preterm birth rates in the year 2010 with time trends since 1990 for selected countries: a systematic analysis and implications. Lancet.

[13] Schmidt L, Sobotka T, Bentzen JG, Nyboe AA (2012). Demographic and medical consequences of the postponement of parenthood. Hum Reprod Update.

[14] Waldenstrom U, Cnattingius S, Vixner L, Norman M (2017). Advanced maternal age increases the risk of very preterm birth, irrespective of parity: a population-based register study. BJOG..

[15] Goisis A, Remes H, Barclay K, Martikainen P, Mikko MM (2017). Advanced maternal age and the risk of low birth weight and preterm delivery: a within-family analysis using Finnish population registers. Am J Epidemiol.

[16] Muniro Z, Tarimo CS, Mahande MJ, Maro E, Mchome B (2019). Grand multiparity as a predictor of adverse pregnancy outcome among women who delivered at a tertiary hospital in northern Tanzania. BMC Pregnancy Childbirth..

[17] McCormick MC (1985). The contribution of low birth weight to infant mortality and childhood morbidity. N Engl J Med.

[18] Kramer MS, Papageorghiou A, Culhane J, Bhutta Z, Goldenberg RL, Gravett M, Iams JD, Conde-Agudelo A, Waller S, Barros F, Knight H (2012). Challenges in defining and classifying the preterm birth syndrome. Am J Obstet Gynecol.

[19] Marlow N, Wolke D, Bracewell MA, Samara M, EPICure Study Group (2005). Neurologic and developmental disability at six years of age after extremely preterm birth. N Engl J Med.

[20] Bureau of National Health Insurance, Taiwan. The National Health Insurance Annual Statistical Report, 2004-2013. Accessed on 15 Aug 2019 by http://www.nhi.gov.tw/00english/e_index.htm.

[21] Delnord M, Blondel B, Prunet C, Zeitlin J (2018). Are risk factors for preterm and early-term live singleton birth the same? A population-based study in France. BMJ Open.

[22] Hernandez-Ibarburu G, Perez-Rey D, Alonso-Oset E, Alonso-Calvo R, de Schepper K, Meloni L, Claerhout B. ICD-10-CM extension with ICD-9 diagnosis codes to support integrated access to clinical legacy data. Int J Med Inform. 2019;129:189–97. 10.1016/j.ijmedinf.2019.06.010 31445254

[23] Wang LK, Chen WM, Chen CP (2014). Preterm birth trend in Taiwan from 2001 to 2009. J Obstet Gynaecol Res.

[24] Chen KH, Chen IC, Yang YC, Chen KT (2019). The trend and associated factors of preterm deliveries from 2001 to 2011 in Taiwan. Medicine (Baltimore).

[25] Morken NH, Källen K, Hagberg H, Jacobsson B (2005). Preterm birth in Sweden 1973-2001: rate, subgroups, and effect of changing patterns in multiple births, maternal age, and smoking. Acta Obstet Gynecol Scand.

[26] Zhang YP, Liu XH, Gao SH, Wang JM, Gu YS, Zhang JY, Zhou X, Li QX (2012). Risk factors for preterm birth in five maternal and child health hospitals in Beijing. PLoS One.

[27] Mgaya AH, Massawe SN, Kidanto HL, Mgaya HN (2013). Grand multiparity: is it still a risk in pregnancy?. BMC Pregnancy Childbirth.

[28] Delbaere I, Verstraelen H, Goetgeluk S, Martens G, De Backer G, Temmerman M (2007). Pregnancy outcome in primiparae of advanced maternal age. Eur J Obstet Gynecol Reprod Biol.

[29] Beck S, Wojdyla D, Say L, Betran AP, Merialdi M, Requejo JH, Rubens C, Menon R, Van Look PF (2010). The worldwide incidence of preterm birth: a systematic review of maternal mortality and morbidity. Bull World Health Organ.

[30] Keiser AM, Salinas YD, DeWan AT, Hawley NL, Donohue PK, Strobino DM (2019). Risks of preterm birth among non-Hispanic black and non-Hispanic white women: effect modification by maternal age. Paediatr Perinat Epidemiol.

[31] Ananth CV, Joseph KS, Oyelese Y, Demissie K, Vintzileos AM (2005). Trends in preterm birth and perinatal mortality among singletons: United States, 1989 through 2000. Obstet Gynecol.

[32] American College of Obstetricians and Gynecologists (2013). ACOG committee opinion no. 561: Non-medically indicated early-term deliveries. Obstet Gynecol.

[33] Barros FC, Velez MP (2006). Temporal trends of preterm birth subtypes and neonatal outcomes. Obstet Gynecol.

[34] Chang YK, Tseng YT, Chen KH, Chen KT (2019). Long-term outcomes and risk factors of thyroid dysfunction during pegylated interferon and ribavirin treatment in patients with chronic hepatitis C infection in Taiwan. BMC Endocr Disord.

[35] MacDorman MF, Reddy UM, Silver RM (2015). Trends in stillbirth by gestational age in the United States, 2006-2012. Obstet Gynecol.

[36] Auger N, Hansen AV, Mortensen L (2013). Contribution of maternal age to preterm birth rates in Denmark and Quebec, 1981-2008. Am J Public Health.

[37] Aldous MB, Edmonson MB (1993). Maternal age at first childbirth and risk of low birth weight and preterm delivery in Washington state. JAMA..

[38] Chan BCP, Tsz-Hsi Lao TTH (2008). Effect of parity and advanced maternal age on obstetric outcome. Int J Gynecol Obstet.

[39] Luke B, Brown MB (2007). Elevated risks of pregnancy complications and adverse outcomes with increasing maternal age. Hum Reprod.

[40] Romero R, Espinoza J, Kusanovic JP, Gotsch F, Hassan S, Erez O, Chaiworapongsa T, Mazor M (2006). The preterm parturition syndrome. BJOG..

[41] Lawn JE, Gravett MG, Nunes TM, Rubens CE, Stanton C (2010). Global report on preterm birth and stillbirth (1 of 7): definitions, description of the burden and opportunities to improve data. BMC Pregnancy Childbirth..

[42] McLernon DJ, Harrild K, Bergh C, Davies MJ, de Neubourg D, Dumoulin JC, Gerris J, Kremer JA, Martikainen H, Mol BW (2010). Clinical effectiveness of elective single versus double embryo transfer: meta-analysis of individual patient data from randomized trials. BMJ..

[43] Jakobsson M, Gissler M, Paavonen J, Tapper AM (2008). The incidence of preterm deliveries decreases in Finland. BJOG..

[44] Hsieh TT, Liou JD, Hsu JJ, Lo LM, Chen SF, Hung TH (2010). Advanced maternal age and adverse perinatal outcomes in an Asian population. Eur J Obstet Gynecol Reprod Biol.

[45] Bayrampour H, Heaman M, Duncan KA, Tough S (2012). Comparison of perception of pregnancy risk of nulliparous women of advanced maternal age and younger age. J Midwifery Womens Health.

[46] Claramonte Nieto M, Meler Barrabes E, Garcia Martínez S, Gutiérrez Prat M, Serra ZB (2019). Impact of aging on obstetric outcomes: defining advanced maternal age in Barcelona. BMC Pregnancy Childbirth..

[47] Liu B, Xu G, Sun Y, Du Y, Gao R, Snetselaar LG, Santillan MK, Bao W (2019). Association between maternal pre-pregnancy obesity and preterm birth according to maternal age and race or ethnicity: a population-based study. Lancet Diabetes Endocrinol.

[48] Langhoff-Roos J, Kesmodel U, Jacobsson B, Rasmussen S, Vogel I (2006). Spontaneous preterm delivery in primiparous women at low risk in Denmark: population based study. BMJ..

[49] Martin JA, Hamilton BE, Sutton PD, Ventura SJ, Menacker F, Kirmeyer S, Munson ML (2007). Births: final data for 2005. Natl Vital Stat Rep.

[50] Engle WA, Tomashek KM, Wallman C, Committee on fetus and newborn, American Academy of Pediatrics (2007). “Late-preterm” infants: a population at risk. Pediatrics..

[51] Bakketeig LS, Hoffman HJ, Harley EE (1979). The tendency to repeat gestational age and birth weight in successive birth. Am J Obstet Gylecol.

[52] Baer RJ, Chambers CD, Ryckman KK, Oltman SP, Rand L, Jelliffe-Pawlowski LL (2019). Risk of preterm and early term birth by maternal drug use. J Perinatol.

[53] Homsup P, Phaloprakarn C, Tangjitgamol S, Manusirivithaya S (2018). Maternal characteristics and pregnancy outcomes among illicit drug-using women in an urban setting. Taiwan J Obstet Gynecol.

[54] Lin HH, Hsu KL, Ko WW, Yang YC, Chang YW, Yu MC, Chen KT (2010). Cost-effectiveness of influenza immunization in adult cancer patients in Taiwan. Clin Microbiol Infect.

